# Spatial-Temporal Variations of Water Quality and Its Relationship to Land Use and Land Cover in Beijing, China

**DOI:** 10.3390/ijerph13050449

**Published:** 2016-04-27

**Authors:** Xiang Chen, Weiqi Zhou, Steward T. A. Pickett, Weifeng Li, Lijian Han

**Affiliations:** 1 State Key laboratory for Urban and Regional Ecology, Research Center for Eco-Environmental Sciences, Chinese Academy of Sciences, No.18 Shuangqing Rd., Haidian District, Beijing 100085, China; xiang8234@126.com (X.C.); li.wf@rcees.ac.cn (W.L.); ljhan@rcees.ac.cn (L.H.); 2 University of Chinese Academy of Sciences, No.19A Yuquan Rd., Beijing 100049, China; 3 Cary Institute of Ecosystem Studies, Box AB, 2801 Sharon Turnpike, Millbrook, NY 12545, USA; picketts@caryinstitute.org

**Keywords:** water quality, land use and land cover, non-point source pollution, scale, Beijing

## Abstract

Rapid urbanization with intense land use and land cover (LULC) change and explosive population growth has a great impact on water quality. The relationship between LULC characteristics and water quality provides important information for non-point sources (NPS) pollution management. In this study, we first quantified the spatial-temporal patterns of five water quality variables in four watersheds with different levels of urbanization in Beijing, China. We then examined the effects of LULC on water quality across different scales, using Pearson correlation analysis, redundancy analysis, and multiple regressions. The results showed that water quality was improved over the sampled years but with no significant difference (*p* > 0.05). However, water quality was significantly different among nonurban and both exurban and urban sites (*p* < 0.05). Forest land was positively correlated with water quality and affected water quality significantly (*p* < 0.05) within a 200 m buffer zone. Impervious surfaces, water, and crop land were negatively correlated with water quality. Crop land and impervious surfaces, however, affected water quality significantly (*p* < 0.05) for buffer sizes greater than 800 m. Grass land had different effects on water quality with the scales. The results provide important insights into the relationship between LULC and water quality, and thus for controlling NPS pollution in urban areas.

## 1. Introduction

Urbanization constitutes high concentrations of urban populations and is an important component of dramatic land transformation [1,2,3]. The changes have great impacts on stream ecosystems, such as the degradation of water quality, simplification of the channel, and decline of biodiversity [4,5,6]. In general, water quality degradation is commonly caused by both non-point sources (NPS) of runoff pollution and point sources (PS) of wastewater discharge. However, with the development of a city and the improvement of wastewater treatment, PS pollution is significantly reduced, and NPS pollution from surface runoff is now becoming a problem for urban water quality [7]. Meanwhile, because human activities differ in intensity in different areas, water quality degradation might be spatially heterogeneous [8,9]. This is particularly perceptible in catchments with environmental land use conflicts [10,11]. Therefore, it is important to analyze the characteristics of river riparian land use/land cover (LULC) and their influence on water quality for NPS pollution management.

There are many studies about the impact of land use/cover on the water quality [7,12,13,14,15,16]. However, the previous studies mainly concentrated on the impacts of the main LULC types of exurban and urban on water quality. In exurban or rural areas, crop and forest land are the primary LULC types; they can increase Nitrogen, Phosphorous, and pesticide concentrations in nearby river water through agricultural activities [17,18,19,20,21,22]. In urban areas, the main LULC type is impervious surfaces. They impact on water quality through higher pollutant concentrations and by increasing the composition of particles, nutrients and metals in runoff waters [23,24,25,26]. Other LULC types, such as water and turf grass, however, have received relatively little attention in research on water quality, although they can also have important effects on water quality. For example, previous studies have shown that percentage cover of water within a catchment had a positive effect on the water quality in a river due to dilution and degradation of pollutants [7]. Grass cover, however, may have a more complex relationship with water quality [19]. The increase of grass coverage was found to increase the concentrations of water pollutants in some case studies [7], but to reduce water pollutants in others [27]. In order to understand the water quality over spatial heterogeneity in large areas, the LULC types which can impact water quality should be analyzed.

The problem of how LULC types influence water quality is very important for water research and management. However, there is controversy regarding to what extent LULC impacts on water quality. Controversy may emerge from the combination of cover class with spatial configuration of the cover. The width of buffer zone beside streams is one case of LULC complexity. For example, 6 m buffer zones composed of trees, shrubs, and grass could reduce total suspended solids by 94%, total nitrogen by 78% and total phosphorus by 81% in runoff from agriculture [28]. Sweeney and Newbold [29] reviewed the literature and found that streamside forest buffer no less than 30 m wide were needed to protect the water quality, aquatic organisms and their habitat in small streams. Houlahan and Findlay [30], however, found that the buffer zone often needs to be nearly 4000 m at the watershed scale to protect the river water quality and the integrity of the aquatic ecological system.

Due to the geographical position and its rapid urbanization, Beijing suffers from a severe lack of water resources and high levels of water pollution. Many cities and regions in the world face the same risks [31]. Therefore, in order to promote sustainable development of the city and enhance human wellbeing, the protection of the aquatic environment is very important. The main objectives of this work are to (1) assess the spatial and temporal pattern of water quality in Beijing; and (2) to investigate the relationships between different types of land and the water quality variables; and (3) to explore the effects of different sizes of buffer zones on the relationship between LULC types and water quality to provide a reliable scientific basis for river management.

## 2. Materials and Methods

### 2.1. Study Area

**Figure 1:**
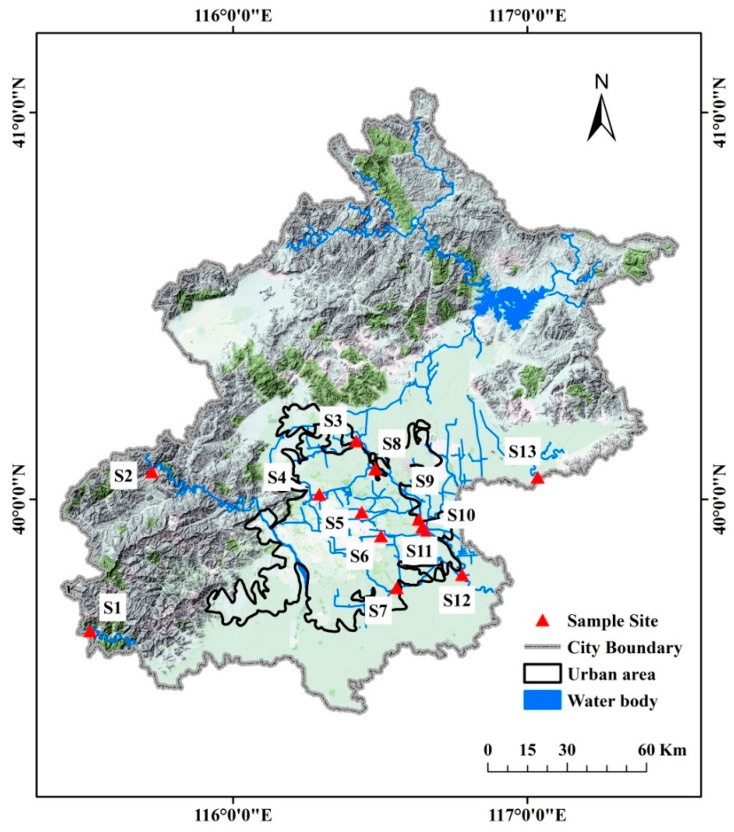
The spatial distribution of the 13 sample sites, overlapping on the topographic map of Beijing (from Google map, 20 July 2015).

This research covers metropolitan Beijing, which is located in the northwest of the North China Plain, adjoining the Inner Mongolian plateau to the north and Taihang Mountains to the west, and covers a total area of 16,410 km^2^. It is located in North Temperate Zone, with hot and rainy summers and cold and dry winters. The annual average temperature is about 12 °C and the annual mean precipitation is 588.1 mm, with about 72% of the precipitation occurring in July and August with <2% occurring in winter. As a politico-commercial and cultural center of China, the population of Beijing was estimated at 21.51 million and the gross domestic product (GDP) was 2133.08 billion RMB in 2014 [32]. Due to geographical contrasts and diverse functional orientations, the development of districts in Beijing is very heterogeneous (Figure 1). In the mountainous areas to the north and west of Beijing, the population density is very low. The degree of urbanization is high in the central plain which is the location of the main concentrations of population, industry and commerce. The northeast plain is far from the city center, and agriculture and subsidiary agricultural products are the main industry and the population is lower than in the central area.

### 2.2. Sites Description and the Water Quality Data

There are five river systems in Beijing. The Beiyun river originates from the local mountains, while the other four river systems, the Chaobai, Jiyun, Yongding, and Daqing, originate from other provinces. There has been no water in the Yongding or the Chaobai downstream since the construction of the Guanting and Miyun reservoirs, respectively. To represent different LULC of the watersheds in this study, 13 sites (S1–S13) are selected based on the established hydrologic monitoring stations (Figure 1). The sites were divided into nonurban, urban and exurban areas according to the watershed area location and the population density [3]. The population proportions of the nonurban, urban and exurban areas, respectively, are about 13:60:27. S1 is in the Juma river, which is a river of the Daqing river system, S2 is located at Yongding upstream, both of these sites are near the mountains and were considered as the nonurban areas. S3–S12 were located on the rivers of the Beiyun river system which has a total length of 89.4 km and an area of 4348 km^2^ in Beijing. The watershed is mainly located in the central urban area, and exhibits the greatest population aggregation. The Beiyun is also the main drainage system in Beijing [7]. S13 is in the Ju river, which is a tributary of the Jiyun river system. It is located in the Pinggu district, about 70 km away from the central city, where human activities are mainly industry, agriculture, and animal husbandry. It is an important source of agricultural and sideline products, and is considered as an exurban area.

Water quality data were obtained from the Beijing Municipal Environmental Monitoring Center, which takes monthly samples of surface water and uses standard methods from the Chinese State Environment Protection Bureau to analyze the water chemistry [33]. Five water quality variables were used because they had relatively complete data records for the study period and they represent important dimensions of water quality. The annual average of the following water quality variables was assessed: dissolved oxygen (DO), ammonium-nitrogen (NH_3_-N), chemical oxygen demand (COD_Cr_), permanganate index (COD_Mn_), and biochemical oxygen demand (BOD). Data of NH_3_-N, COD_Mn_, and BOD were from 2000 to 2010. Data of DO and COD_Cr_ were from 2005 to 2010.

### 2.3. Land Use and Land Cover (LULC) Data

**Figure 2:**
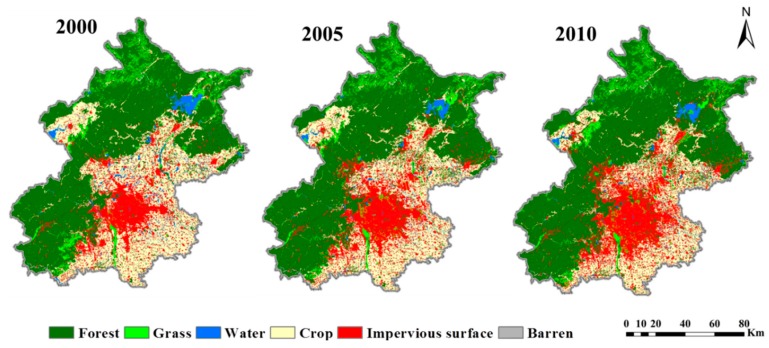
Land use and land cover of Beijing in 2000, 2005, and 2010.

Land use and land cover data were obtained from classification of Landsat 5 Thematic Mapper (TM) images collected in 2000, 2005, and 2010, with a spatial resolution of 30 m. These images obtained from United States Geological Survey (USGS), with the primary process though Level 1 Product Generation Survey (LPGS). In this pre-processing, systematic radiometric and geometric corrections were performed [34]. In order to improve the classification accuracy for the LULC map of 2005 and 2000, we further normalized the 2005 and 2000 TM image using 2010 TM data as the reference. Six LULC types were included, that is, impervious surface, crops, forest, grass, water, and barren. We combined an object-based classification approach with a backdating method for classifications. Specifically, classifications were first conducted on the images for 2010 using an object-based classification approach [35]. We then used the LULC data of 2010 as the reference, and applied a backdating approach to classify the images for 2000 and 2005 [36,37] (Figure 2). The overall accuracies of the classifications were 94%, 93% and 94% [38], respectively.

### 2.4. Construction Buffers in Urban Rivers

We used contrasting buffer scales in this research, as previous studies have shown that the relationship between LULC and water quality was similar for the catchment scale and the scale of a 100 m buffer [12]. To quantify the effects the different LULC types might have on river water quality at different distances from the river, we reference another method used by researchers [39] and created a wide range of buffer zones (100 m, 200 m, 300 m, 400 m, 500 m, 600 m, 800 m, 1000 m, 1200 m, 1500 m and 2000 m) for the sites using ArcGIS 10.1 (Environmental Systems Research Institute, Inc., Redlands, CA, USA). The urban stream syndrome showed that the interactions between streams and catchment are more complex in urban areas than natural systems because of the pipe drainage network [5]. To understand the relationship between water quality and land use in an urbanizing watershed, Sonoda *et al.* quantified percentages of different land use types within radius sizes of 30 m, 90 m and 152 m at each site [24]. In this research, the length range of 1000 m upstream and 100 m downstream of each site was identified based on the first grade of classifying water source reserves in rivers [40], and the LULC types in buffers of the river section were used to calculate the relationship with water quality variables.

### 2.5. Statistical Analysis

**Figure 3:**
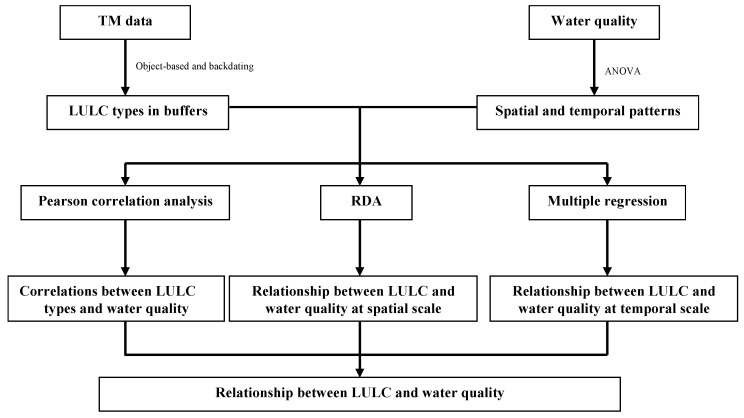
Framework of the methods used for analysis the relationships of LULC and water quality.

One-way analysis of variance (ANOVA) was used to test the significance of the differences among the water quality variables from the three areas. Paired *t*-test was performed to test for significant differences of the DO and COD_Cr_ between 2005 and 2010. Pearson correlation analysis was used to investigate the quantitative relationship between the LULC types and water quality variables in buffer zones at spatial scales in 2000, 2005, and 2010 (Figure 3). Redundancy analysis (RDA) was employed to identify the LULC types and patterns that impact the water quality variables across a series of spatial scales at different sites [41]. Before using the RDA, detrended correspondence analysis (DCA) was used to explore the length of the gradient of the four ordination axes before selecting the appropriate model in CANOCO 4.5 (Microcomputer Power, Ithaca, NY, USA). The longest gradient of the four ordination axes was less than 3 in all buffer zones, and the linear model of RDA was selected. From spatial and temporal scales, multiple regression analysis was used to test the correlations of LULC types within buffers and water quality in 2000, 2005, and 2010. In the analysis, water quality variables were treated as dependent variables and the percentage cover of LULC types were considered as independent variables. We used a conventional 5% level of significance to test the statistical significant difference in all statistical analyses with SPSS 19.0 (IBM, New York, NY, USA) for Windows. DCA and RDA were performed to use CANOCO 4.5 for Windows [41].

## 3. Results

### 3.1. Spatial and Temporal Pattern of Water Quality

**Figure 4:**
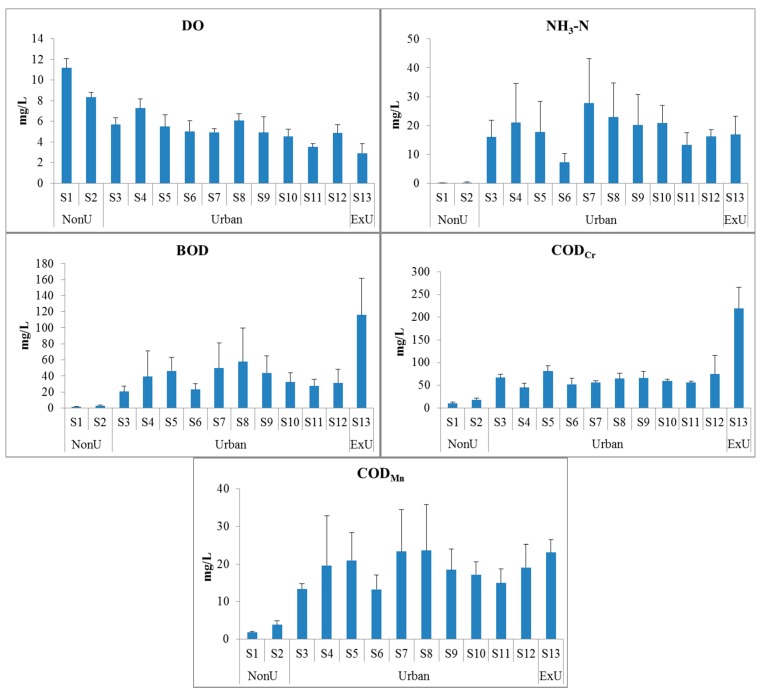
Spatial patterns of water quality in Beijing. The bars were the annual average concentrations for each station. “NonU” means nonurban sites; “ExU” means exurban sites.

The results showed that water quality in the study area had significant spatial variation. Water quality in nonurban area was better than the exurban and urban areas, and the exurban water quality was the worst. Concentrations of water quality variables were significantly higher (*p* < 0.01) in the exurban area than in the urban area except for NH_3_-N, and in both areas water quality variables were significantly different (*p* < 0.001) from nonurban areas (Figure 4). For example, the mean concentration of BOD in the exurban area was 115.8 mg/L, higher than the 37.1 mg/L in the urban area and the mean of 2.22 mg/L in the nonurban area. The concentration of COD_Cr_ in the exurban area was 220 mg/L, also higher than 62.4 mg/L in the urban area and 14.1 mg/L in the nonurban area. However, the concentration of DO in nonurban area was 9.77 mg/L, higher than 5.24 mg/L in the urban area and 2.92 mg/L in the exurban area.

During the period of 2000–2010, water quality improved in Beijing, especially from 2005 to 2010 (Table 1). In urban areas, for example, the concentration of COD_Mn_ decreased from 22.89 mg/L in 2000, to 18 mg/L in 2005, and to 11.72 mg/L in 2010 (*p* < 0.01). BOD decreased from 58.82 mg/L in 2000, to 34.01 mg/L in 2005 (*p* < 0.05), and to 22.39 mg/L in 2010 (*p* < 0.01). Concentration of NH_3_-N and COD_Cr_ also decreased in urban areas, but the concentration of DO increased from 4.87 mg/L in 2005 to 5.16 mg/L in 2010 (*p* > 0.05). In exurban areas, the concentration of NH_3_-N, COD_Cr_, and COD_Mn_ decreased from 2005 to 2010, and the concentration of DO increased from 2005 to 2010, however, the concentration of BOD increased from 2000 to 2010. In nonurban areas, the concentration of NH_3_-N, BOD, and COD_Mn_ showed a fluctuating trend in 2000, 2005 and 2010. For example, the concentration of BOD decreased from 2.25 mg/L in 2000 to 1.9 mg/L in 2005, but increased to 2.35 mg/L in 2010, which was higher than 2000. COD_Mn_, however, the concentration increased from 1.95 mg/L in 2000 to 3.15 mg/L in 2005, but declined to 2.93 mg/L in 2010.

### 3.2. Spatial Patterns of LULC in Buffers

**Figure 5:**
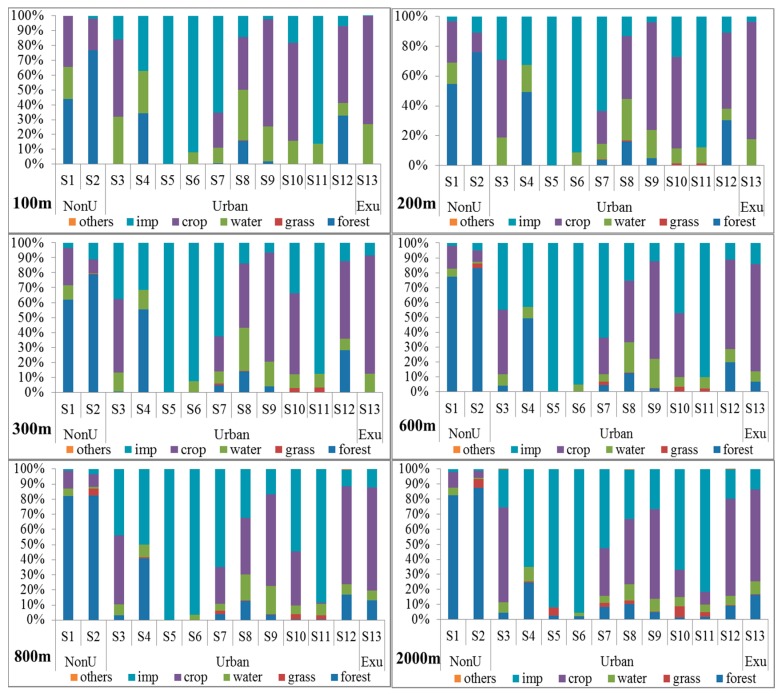
LULC characteristics of the river section within the buffers of 100 m, 200 m, 300 m, 600 m, 800 m, and 2000 m. “imp” means impervious surface.

The proportion of the LULC types changed with an increase in the buffer zones from 100 m to 2000 m (Figure 5). Within the nonurban areas, as the buffer zones increased, the percentage of forest land increased from 44% to 82% in S1 and from 76% to 87% in S2, and the percentage of crop land and impervious surface decreased accordingly. The sites of S4, S5, S6, S7, and S11 were in the central urban area, and the mean percentage of impervious surface was about 70%. Especially in S5 and S6, the percentage of impervious surface was over 90% from 100 m to 2000 m, and the percentage of impervious surface was over 80% in S11 from 100 m to 2000 m. The sites of S3, S9, S10 and S12 were near the central urban area, and the percentage of impervious surface was lower than the central urban area sites. However, the percentage of impervious surface increased with the increase of buffer zone. For example, the percentage of impervious surface increased from 7% in 100 m to 19.5% in 2000 m in S12. Crop land was the main LULC type in the exurban area and the mean percentage of crop land was 70.2% from 100 m to 2000 m in S13. However, the percentage of crop land decreased from 72% in 100 m to 61% in 2000 m, but the percentage of forest land and impervious surface increased accordingly. Percentage of grass land in the sampling sites was lower than other LULC types. For example, the percentage of grass land was 0.5% in 100 m and 2.4% in 2000 m in S8.

### 3.3. Relationship between Water Quality and LULC in Buffers

#### 3.3.1. Results of Pearson Relationship Analysis

**Figure 6:**
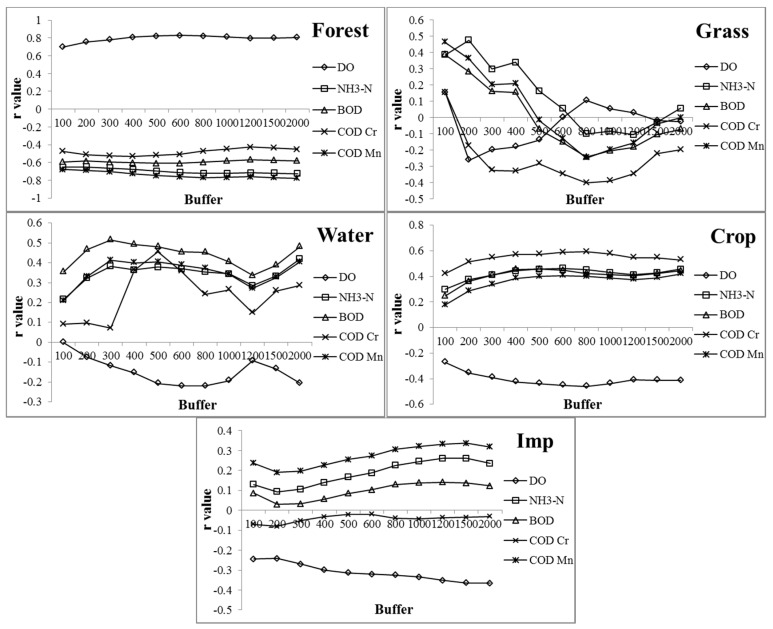
Pearson relationships between the LULC types of Forest, Grass, Water, Crop, and Imp (impervious surface) and water quality variables within the buffer of 100–2000 m.

Calculation of the Pearson correlation coefficient showed that the LULC types have very different impacts on water quality (Figure 6). Forest land was positively correlated with water quality. Pearson results showed that the forest land was positively correlated with DO, but negatively correlated with NH_3_-N, BOD, COD_Cr_ and COD_Mn_ from 100 m to 2000 m. However, the LULC types of water, crop, and impervious surface were negatively correlated with water quality. The LULC types were negatively correlated with DO, but positively correlated with NH_3_-N, BOD, COD_Cr_ and COD_Mn_ except for impervious surface, which was negatively correlated with COD_Cr_ from 100 m to 2000 m. Pearson results demonstrated that grass land has a different correlation with water quality when the width of the buffer zone changed. Grass land was positively correlated with NH_3_-N, BOD, and COD_Cr_, but negatively correlated with DO from 100 m to 500 m; however, it was negatively correlated with NH_3_-N, BOD, and COD_Cr_ from 600 m to 2000 m.

Correlation coefficients between LULC types and water quality variables changed when the buffer zones increased. In the case of water, for example, the correlation coefficients with COD_Cr_ were less than 0.1 in the first 300 m and increased to 0.456 at 500 m, then decreased to 0.148 at 1200 m, and increased to 0.286 at 2000 m. However, with DO, the correlation coefficients increased continually from 0.001 at 100 m to 0.219 at 800 m, but decreased from 800 m to 1200 m, then increased back to 0.206 at 2000 m. The correlation coefficients between water and NH_3_-N, BOD, and COD_Mn_ were similar to DO, but decreased earlier from 300 m to 1200 m. The correlation coefficients of impervious surface with NH_3_-N, BOD, and COD_Mn_ decreased from 100 m to 200 m, but increased continually to 1500 m, then decreased back to 2000 m. However, the correlation coefficient with DO increased continually from 0.244 at 100 m to 0.365 at 2000 m. The correlation coefficients of crop land with the five water quality variables increased from 100 m to 2000 m, but the correlation coefficients of forest with the water quality variables changed less than other LULC types.

The results also showed that the main water quality variable affected by LULC type was very different. For example, the correlation coefficient of forest with DO was higher than other water quality variables, but the correlation coefficient of forest with COD_Cr_ was minimal for the water quality variables. The correlation coefficient of water with BOD was higher than other water quality variables, but lower with DO than other water quality variables. The correlation coefficient of crop land with COD_Cr_, however, was higher than other water quality variables, but impervious surface had a higher correlation coefficient with COD_Mn_.

#### 3.3.2. Results of Redundancy Analysis

**Figure 7:**
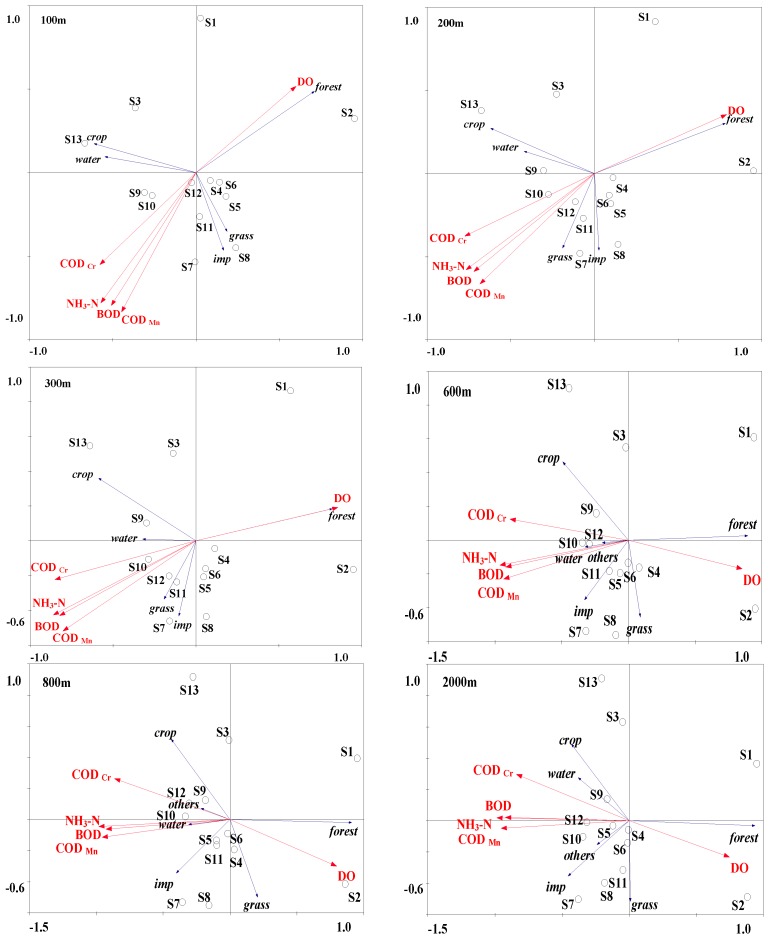
RDA results of LULC and water quality variables within the buffer of 100 m, 200 m, 300 m, 600 m, 800 m, and 2000 m. “imp” means impervious surface.

Redundancy analysis (RDA) showed the correlations of water quality variables and LULC types in the sites along with the different buffer zones (Figure 7). The water chemistry variables were mainly explained by the first two axes, and the first axis explained the variables more than twice as well as the second axis (Table 2). The first axis was positively correlated with forest land and negatively correlated with crop land and impervious surface, which increased in the urban and exurban area. The second axis was positively correlated with crop land and negatively with urbanization. Although the ordination results showed similar interactions to the results of Pearson analyses, the relationship between LULC and the water chemistry variables could be very clearly understood using ordination. From 100 m to 2000 m, DO was positively correlated with forest, which was the main LULC type in nonurban area sites, S1 and S2.

#### 3.3.3. Results of Multiple Regression Analysis

The results of multiple regression analysis indicated that buffer sizes were very important for studying the relationship between LULC types and water quality (Table 3). At buffers of 100 m and 200 m, forest land was an important factor for water quality over the years. For example, the forest was the first LULC type correlated with the water quality indicators, and it was very significantly negatively correlated with COD_Mn_ (Adjust *R*^2^ = 0.516, *p* < 0.01), NH_3_-N (Adjust *R*^2^ = 0.629, *p* < 0.01) in 2010, and significantly negatively correlated with BOD (*R*^2^ = 0.191, *p* < 0.05)), COD_Cr_ (*R*^2^ = 0.362, *p* < 0.05) in 2010, but significantly positively correlated with DO (*R*^2^ = 0.593, *p* < 0.01) at the 100 m buffer. However, at the 300–600 m buffers, the mixed types of crop, impervious surface, water, and forest all significantly affected water quality. AT the buffer of 300 m, the COD_Mn_ (*R*^2^ = 0.634, *p* < 0.05), BOD (*R*^2^ = 0.63, *p* < 0.01), and NH_3_-N (*R*^2^ = 0.361, *p* < 0.05) were significantly correlated with water land in 2000; but all of the water quality indicators were significantly correlated with crop land in 2010; and forest land was the first LULC type correlated with the water quality indicators in 2005. From 800 m to 2000 m, however, crop land and impervious surfaces were the main factors affecting water quality. The crop and impervious surfaces were significantly correlated with COD_Mn_ (Adjust *R*^2^ = 0.462, *p* < 0.05), and NH_3_-N (Adjust *R*^2^ = 0.845, *p* < 0.001) in 2010, and significantly correlated with DO in 2005 (Adjust *R*^2^ = 0.585, *p* < 0.01) and 2010 (Adjust *R*^2^ = 0.587, *p* < 0.01).

## 4. Discussion

Surface water can be polluted by point source pollution (PS) and non-point sources of pollution (NPS) [7,17]. With the development of wastewater treatment technology and the increased attention of the government, point source pollution is increasingly controlled in Beijing. For example, the Beijing urban waste treatment rate reached 85% in 2014, an increase from the 40% treatment rate in 2000 [32,42]. However, although PS pollution has been better controlled, the water quality indexed by five variables did not improve significantly, especially in exurban areas (Table 1). Therefore, untreated NPS pollution may continue to be an important factor affecting the water quality in Beijing (see also [7]). NPS pollution is considered to be another main factor influencing water quality as well as the PS polluted wastewater without treatment [43,44].

The water quality of Beijing differed greatly according to location (Figure 4), and LULC types correlated well with water chemistry variables (Figure 5 and Figure 6). All of the LULC types, such as impervious surfaces, crop land and forest land, had significant effects on water quality (Table 3). From 2000 to 2010, the area of impervious surface cover increased from 2186 km^2^ to 2964 km^2^ in Beijing; the rapid increase of impervious surfaces certainly will impact nearby water quality. The Pearson correlation analyses showed that impervious surface was positively correlated with NH_3_-N, BOD, and COD_Mn_ in different buffer zones. Also, with the impervious surface area increasing, the relationship between the impervious surface and water quality become more significant. For example, at the buffer of 100 m, the coefficient of impervious surface and water quality was greater in 2010 than 2000 and 2005. As the proportion of impervious surfaces increased across sites, surface runoff increased with storm water, which is one of the main sources of water pollution in urban areas [12,23,45,46].

The area of agricultural crops was also positively correlated with COD_Mn_, BOD and NH_3_-N, and had the strongest correlation with COD_Cr_ (*r* > 0.5, *p* < 0.05). In the exurban site, crop land was the main LULC type. The impact of agricultural non-point source pollution on water quality has received much attention, and many studies report that agricultural activities are strongly correlated with high nutrients in streams, such as nitrogen and phosphorus, along with pesticides [12,20,47]. In the course of agricultural activities, large amounts of organic pesticides and fertilizers are applied to crop land. Most organic pesticides are hard to degrade and residues run into nearby rivers with heavy rain and irrigation. Such contamination could lead to the high COD and concentration of other pollutants in the water [18,48]. In addition, there are many livestock farms in the Beijing exurban area. Due to a shortage of treatment plants, most of the waste directly enters local waterways. In order to protect the water quality in exurban areas, nutrient management, such as nutrient budget, is a mitigation measure to diffuse water pollution from agriculture [49]. Zheng *et al.* [50] also suggested that payment for ecosystem services (PES) would be an important measure to reduce output from agricultural activities.

The LULC type of water negatively correlated with water quality. Though the waste treatment rate reached 85% in Beijing by 2014, the untreated waste waters are still a relatively large pollution source. By 2014, water quality of 53.1% of the rivers in Beijing was poor or very poor [51]. In urban river systems, such as the Beiyun river system, the poor and very poor water quality is more than 80%, while in exurban areas the proportion is nearly 70%. This means the water quality in most urban and exurban river sections is poor, and the reaches upstream of sample stations have no effect on the purification of downstream water. Beijing is characterized by a severe water shortage, and rainfall is the major water source for its rivers. However, rainfall is also a source of pollution in Beijing because of storm runoff. In urban areas, the storm runoff contains a mixture of pollutants from roofs and roads, which feed into the rivers, lakes and wetlands [27,52]. Indeed, lakes and wetlands are the main water source of rivers in dry seasons in the central city area of Beijing. Some researchers believe the LULC type of water should be negatively correlated with water quality parameters because of dilution and purification [27]. However, the water they referred to was reclaimed water from wastewater treatment plants (WWTPs), which has relatively low pollutant concentrations compared with storm runoff water [53].

We found forest land was the only land cover type that is negatively related to pollutants and positively correlated with DO, reflecting its positive role in controlling water quality by filtration, absorption and translation of pollutants before they run into waterways [54,55,56,57]. For example, Dosskey *et al.* [55] reviewed research on how riparian vegetation affected water quality, and found that the vegetation could take up 170 kg nitrogen and 49 kg phosphorous per ha every year. Multiple regression analysis demonstrated that forest land in Beijing was the primary factor affecting water quality at a 200 m buffer. This may be associated with how river management in Beijing is conducted. In order to improve the urban environment, the government constructed green ways on both sides of 10 major rivers and some parks in the center city. By 2017, a projected 1000 km of green way will be built in Beijing, which is believed to benefit people, ecosystems and habitats, and assist in water provision [58]. Bernhardt and Palmer [59] believe that riparian forest is important for maintaining high water quality in stormwater management and stream restoration. Our results suggest that planting forests beside the banks of urban streams will greatly improve the water quality by controlling NPS pollution in Beijing.

Some research has suggested that grass land can also promote water quality [14,60,61], whereas other studies showed that the increasing proportion of grass land was strongly associated with water quality degradation [19,62]. Our results, however, showed that the effect of grass land on water quality was sensitive to spatial scale. At buffers of 500 m, the grass land was positively correlated with pollutants of COD_Mn_, BOD, and NH_3_-N, but from 600 m to 2000 m, it was negatively correlated with these pollutants. Planting grass to protect the slope on river banks resulted in intensive management measures, such as watering, fertilizing, spraying insecticide and clipping grass, which would have a negative impact on water quality [27]. However, as the buffer size increases, the planted and highly managed grass land was replaced by minimally managed grass land, which was affected only little by humans. Such lightly managed grass land can intercept some pollutants in rain water that is on its way to rivers [61].

The scale effect was an important factor in the relationship between LULC and water quality but the range remains controversial. For example, Shen *et al.* [39] found landscape metrics could explain 73% of the water quality variation at 300 m, but the explanatory power decreased to 64% at 500 m. Zhou *et al.* [63] found the LULC pattern had a major impact on water quality in assessing the landscape pattern effects on water quality at scales from 500 m to 5000 m. Tu [16] explored the relationship between water quality and land use at a regional scale from 20 km to 80 km away from the metropolitan Boston area, and showed that land use explained water quality variation across the urbanization gradient in the watersheds. Our results indicated that the forest land was the most important LULC type to affect water quality at 200 m buffers, but the crop land and impervious surfaces were the primary LULC types effecting water quality at buffers greater than 800 m.

The distribution of wastewater treatment plants (WWTPs) was another important factor in the spatial difference of water quality in Beijing. Our results showed that the pollutant concentrations were very high at the urban sampling stations, but the concentration of pollutants at the exurban station was higher than in the urban area (Table 1). The results demonstrated that the exurban river water quality was worse than that in the urban area, and the public and the government should pay more attention to the exurban river environment. For example, there should be more monitoring stations and more WWTPs in the exurban area. By 2010, there was a total of 60 WWTPs in Beijing, and nearly 80 percent of them are in urban areas. There are only 16 WWTPs capable of processing more than 50,000 tons per day and 13 of them are distributed in the urban area; the other three WWTPs are scattered in different areas. Pinggu district, for example, the most important producer of agricultural and subsidiary products in Beijing, has only three WWTPs and just one plant with a processing capacity of about 80,000 tons per day. The other two WWTPs in Pinggu have processing capacities of only 1500 tons per day. Most untreated wastewater is discharged into rivers and degrades the water quality. Lack of WWTPs and the shortage in processing capacity in the exurban and rural areas are a big problem faced by most cities in China [20,64]. Therefore, it is important to build WWTPs in exurban and rural areas to improve the water quality and people’s living environment.

## 5. Conclusions

We analyzed the spatial and temporal patterns of water quality in Beijing. Our results showed that water quality was significantly different in areas exhibiting different degrees of urbanization, and that LULC was an important factor in determining water quality. In addition we documented that forest areas have a positive relationship with water quality. In contrast, other LULC types, such as impervious surfaces, crops, and water, were negatively related with water quality. The grass land, however, had differing effects on water quality because of different intensities of grass land management at wider buffers. The grass land near rivers was mainly used to protect the slope, and it may have negative impacts on water quality as a result of intensive management. However, as the buffer size increases, the managed grass land near the river bank is replaced by the natural grass land, which may have a positive effect on water quality by retaining pollutants from storm water. Accordingly, we believe that less fertilizing and spraying of insecticide, decreasing artificial grass land, and keeping the grass land in a more natural state are better strategies than intensive management within buffer zone to ensure water quality.

Our results also showed that buffer size was an important factor affecting water quality. Within the 200 m buffer zone, forest land was the most important LULC type affecting water quality, and it was positively correlated with water quality. However, forest, crop, and water were the most important types affecting water quality from 300 m to 800 m. As the buffer zone increased to 800 m, crop land and impervious surfaces gradually became the main LULC types affecting water quality, and they were negatively correlated with water quality. Finally, we suggest increasing the proportion of forest land in the buffer zones with minimally managed grass land near the river in urban areas, and maintaining the natural characteristics in nonurban areas as well as preventing the development of the river bank in exurban areas may all be important for controlling NPS pollution.

## Figures and Tables

**Table 1:** Mean value of water quality variables at nonurban areas, and urban areas and exurban areas in 2000, 2005, and 2010.

Variables	Nonurban Area			Urban Area			Exurban Area		
	2000	2005	2010	2000	2005	2010	2000	2005	2010
DO		9.89	8.69		4.87	5.16		2.09	2.62
NH_3_-N	0.13	0.24	0.24	19.38	16.66	12.25	9.7	29.3	16.6
BOD	2.25	1.9	2.35	58.82	34.01	22.39	29.7	81.8	99.7
COD_Cr_		17.35	12.6		70.11	63.55		229	174
COD_Mn_	1.95	3.15	2.93	22.89	18	11.72	20	25.1	23.4

**Table 2:** Summary of RDA of LULC with respect to the five environmental variables (ENVI) at buffers of 100–2000 m.

Buffer (m)	Axis 1			Axis 2			Total Variance (%)
	Correlations of LULC-ENVI	% Variance of		Correlations of LULC-ENVI	% Variance of		
		LULC	LULC-ENVI		LULC	LULC-ENVI	
100	0.815	22.7	55.3	0.514	16.2	39.6	41.1
200	0.878	26.1	63.3	0.468	14	34	41.2
300	0.895	28.8	68.1	0.452	12.6	29.9	42.3
400	0.892	31.6	72.3	0.445	11.7	26.7	43.7
500	0.891	33.7	74.9	0.442	10.9	24.4	45
600	0.897	35.5	76.2	0.447	10.8	23.1	46.6
800	0.889	37.7	75.8	0.48	11.7	23.4	49.7
1000	0.882	38.3	75	0.503	12.6	24.5	51.1
1200	0.875	38.8	74.6	0.518	13	24.9	52
1500	0.894	40.9	75.4	0.525	13.2	24.3	54.3
2000	0.917	41.9	78.2	0.483	11.5	21.6	53.6

**Table 3:** Summary of multiple regression analysis between LULC types in 100 m, 300 m, and 2000 m buffer sizes and water quality variables. We list the three most important LULC variables, based on their standardized coefficients (Std. Coef.).

Buffer (m)	Variables	First		Second		Third		*R*^2^ Adj
		LULC	Std. Coef.	LULC	Std. Coef.	LULC	Std. Coef.	
100	COD_Mn__2000	water	0.516	forest	−0.406	imp	0.341	0.463
	COD_Mn__2005	forest	−0.581	water	−0.114	imp	−0.103	0.043
	COD_Mn__2010	forest	−1.053 **	imp	−0.589	grass	0.327	0.516
	BOD_2000	water	0.792 *	imp	0.421	forest	−0.113	0.423
	BOD_2005	forest	−0.529	imp	−0.18	water	−0.134	−0.055
	BOD_2010	forest	−0.861 *	imp	−0.641	grass	0.229	0.191
	NH_3_-N_2000	water	0.542	imp	0.338	forest	−0.213	0.187
	NH_3_-N_2005	forest	−0.657	imp	−0.237	water	−0.097	0.101
	NH_3_-N_2010	forest	−1.05 **	imp	0.542	grass	0.259	0.629
	COD_Cr__2005	forest	−0.561	imp	−0.437	water	−0.162	−0.022
	COD_Cr__2010	forest	−0.975 *	imp	−0.529	grass	0.261	0.031
	DO_2005	forest	0.777 *	imp	0.214	water	0.124	0.298
	DO_2010	forest	1.075 **	water	0.514 *	imp	0.425	0.593
300	COD_Mn__2000	water	0.72 *	imp	0.392	forest	−0.344	0.634
	COD_Mn__2005	forest	−0.54	grass	0.286	imp	−0.101	0.03
	COD_Mn__2010	crop	0.999 **	imp	0.672 *	water	0.181	0.552
	BOD_2000	water	0.94 **	imp	0.418	forest	−0.071	0.63
	BOD_2005	forest	−0.44	grass	0.419	imp	−0.123	0.038
	BOD_2010	crop	0.87 *	imp	0.431	water	0.102	0.285
	NH_3_-N_2000	water	0.811 *	imp	0.459	grass	0.318	0.361
	NH_3_-N_2005	forest	−0.563	grass	0.499	imp	−0.158	0.347
	NH_3_-N_2010	crop	0.942 **	imp	0.742 **	water	0.387 *	0.7
	COD_Cr__2005	forest	−0.691	imp	−0.502	grass	−0.294	0.092
	COD_Cr__2010	crop	0.967 **	imp	0.707 *	water	0.115	0.446
	DO_2005	forest	0.844 *	imp	0.24	water	0.093	0.343
	DO_2010	crop	−1.025 ***	imp	−0.872 **	grass	−0.254	0.751
2000	COD_Mn__2000	water	0.65 *	forest	−0.358	grass	0.261	0.606
	COD_Mn__2005	crop	0.798	imp	0.671	water	−0.14	0.21
	COD_Mn__2010	crop	0.933 **	imp	0.56 *	grass	0.326	0.462
	BOD_2000	water	0.966 *	imp	0.36	grass	0.359	0.537
	BOD_2005	crop	0.658	imp	0.376	grass	0.091	−0.121
	BOD_2010	crop	0.595	imp	0.233	grass	0.06	−0.09
	NH_3_-N_2000	water	0.91 **	grass	0.446	imp	0.381	0.669
	NH_3_-N_2005	crop	0.788	imp	0.523	grass	0.145	0.081
	NH_3_-N_2010	crop	1.09 ***	imp	0.655 ***	grass	0.45 **	0.845
	COD_Cr__2005	crop	0.7	imp	0.24	grass	0.145	0.013
	COD_Cr__2010	crop	0.741	imp	0.465	grass	0.078	0.076
	DO_2005	crop	−0.939 **	imp	−0.827 **	grass	−0.324	0.585
	DO_2010	crop	−0.974 **	imp	−0.798 **	grass	−0.265	0.587

* Coefficient is significant at the 0.05 level (two-tailed); ** Coefficient is significant at the 0.01 level (two-tailed); *** Coefficient is significant at the 0.001 level (two-tailed). “imp” means impervious surface.

## Supplementary Files

Supplementary File 1
